# The statistical approach in trial-based economic evaluations matters: get your statistics together!

**DOI:** 10.1186/s12913-021-06513-1

**Published:** 2021-05-19

**Authors:** Elizabeth N. Mutubuki, Mohamed El Alili, Judith E. Bosmans, Teddy Oosterhuis, Frank J. Snoek, Raymond W. J. G. Ostelo, Maurits W. van Tulder, Johanna M. van Dongen

**Affiliations:** 1 Department of Health Sciences, Faculty of Science, Vrije Universiteit Amsterdam, Amsterdam Movement Sciences Research Institute, Amsterdam, the Netherlands; 2 Department of Epidemiology and Biostatistics, Amsterdam UMC, Vrije Universiteit Amsterdam, Amsterdam Movement Sciences Research Institute, Amsterdam, the Netherlands; 3 Department of Health Sciences, Faculty of Science, Vrije Universiteit Amsterdam, Amsterdam Public Health Research Institute, Amsterdam, the Netherlands; 4 Department of Medical Psychology, Amsterdam UMC, Vrije Universiteit Amsterdam, Amsterdam, the Netherlands; 5 Department of Physiotherapy & Occupational Therapy, Aarhus University Hospital, Aarhus, Denmark; 6 Department of Human Movement Sciences, Faculty of Behavioural and Movement Sciences, Vrije Universiteit Amsterdam, Amsterdam, the Netherlands

**Keywords:** Cost-benefit analysis, Statistical methods, Clinical trial, Missing data, Skewed data, Baseline imbalances

## Abstract

**Background:**

Baseline imbalances, skewed costs, the correlation between costs and effects, and missing data are statistical challenges that are often not adequately accounted for in the analysis of cost-effectiveness data. This study aims to illustrate the impact of accounting for these statistical challenges in trial-based economic evaluations.

**Methods:**

Data from two trial-based economic evaluations, the REALISE and HypoAware studies, were used. In total, 14 full cost-effectiveness analyses were performed per study, in which the four statistical challenges in trial-based economic evaluations were taken into account step-by-step. Statistical approaches were compared in terms of the resulting cost and effect differences, ICERs, and probabilities of cost-effectiveness.

**Results:**

In the REALISE study and HypoAware study, the ICER ranged from 636,744€/QALY and 90,989€/QALY when ignoring all statistical challenges to − 7502€/QALY and 46,592€/QALY when accounting for all statistical challenges, respectively. The probabilities of the intervention being cost-effective at 0€/ QALY gained were 0.67 and 0.59 when ignoring all statistical challenges, and 0.54 and 0.27 when all of the statistical challenges were taken into account for the REALISE study and HypoAware study, respectively.

**Conclusions:**

Not accounting for baseline imbalances, skewed costs, correlated costs and effects, and missing data in trial-based economic evaluations may notably impact results. Therefore, when conducting trial-based economic evaluations, it is important to align the statistical approach with the identified statistical challenges in cost-effectiveness data. To facilitate researchers in handling statistical challenges in trial-based economic evaluations, software code is provided.

**Supplementary Information:**

The online version contains supplementary material available at 10.1186/s12913-021-06513-1.

## Highlights

- Previous research shows that the statistical quality of many trial-based economic evaluations is poor.
- This study evaluates the impact of simultaneously accounting for various statistical challenges on the results of trial-based economic evaluations.
- Ignoring these statistical challenges in trial-based economic evaluations may notably impact the results.
- It is important to avoid misalignment between data characteristics and the statistical approach.
- To facilitate researchers in handling statistical challenges in trial-based economic evaluations, software code (Stata®) is provided.

## Introduction

Economic evaluations aim to inform resource allocation decisions in healthcare by evaluating whether the additional health benefits of an intervention justify its additional costs [1]. In many countries, economic evaluations are increasingly being accepted as a formal decision criterion for the reimbursement of pharmaceuticals and other healthcare technologies [2, 3]. Consequently, there is growing interest in economic evaluations of healthcare interventions.

Although great improvements in the conduct and reporting of economic evaluations along clinical trials have been made in previous years [4], literature shows that the quality of the applied statistical methods is typically far from optimal [4–6]. Often, baseline imbalances, the skewed nature of cost data, and the correlation between costs and effects are not adequately accounted for [7, 8]. Additionally, missing data are frequently handled using “naïve” imputation methods, such as mean imputation and last observation carried forward [7–11]. Failure to appropriately account for these statistical issues when analyzing trial-based economic evaluations is of great concern, because use of inadequate statistical methods may lead to biased results, and consequently invalid decisions resulting in a potential waste of scarce resources [4].

In recent years, various studies have been performed on how to deal with baseline imbalances, skewed costs, correlated costs and effects, and missing data in trial-based economic evaluations [7, 9, 10, 12–17]. A more detailed description of these statistical issues and how to deal with them can be found in Table 1 [51]. Although previous studies have investigated how to deal with these statistical challenges in trial-based economic evaluations separately, the impact of simultaneously accounting for these statistical challenges on the results of trial-based economic evaluations has not yet been explored. The current study aims to address this gap in knowledge, by analyzing data from two previous trial-based economic evaluations, the REALISE and HypoAware studies, whilst step-by-step taking into account the aforementioned statistical challenges in the analysis of the data.

**Table 1:** Statistical challenges in trial-based economic evaluations

*1). Baseline imbalances*	
It is commonly assumed that the random allocation of participants in trial-based economic evaluations ensures that observed and non-observed characteristics are well-balanced across study groups. Nevertheless, some between-group differences in baseline values and/or important prognostic factors regularly occur [18]. Failure to account for such baseline imbalances will likely lead to biased results [18, 19]. Various methods have been suggested to account for baseline imbalances in trial-based economic evaluations, including mean difference adjustment, regression-based adjustment, and matching methods [13, 15, 18–20]. In the literature, regression-based approaches are most commonly used to deal with baseline imbalances. Advantages of regression-based adjustment are that it enables to adjust for various covariates simultaneously, its ability to identify important subgroups by using interaction terms, and its relatively simple implementation in standard statistical software packages [13, 15, 18, 20–26]. However, regression-based adjustment also has a number of drawbacks, including the need for normally distributed residuals and similar distributions of covariates across treatment arms [15]. Moreover, adjusting for several covariates simultaneously might result in overfitting of the model which leads to misleading goodness-of-fit statistics, regression coefficients and *p*-values. In addition, matching methods can be used, which include propensity score adjustment and propensity score matching. However, these methods are specifically developed for trial-based economic evaluations that use non-randomized study designs, because of their ability to deal with the non-randomized nature of data.	
*2) Skewed costs*	
Costs are generally right-skewed as there are relatively few participants with (very) high costs and it is impossible to incur negative costs. Consequently, the assumption of normality of standard parametric tests, such as t-tests and linear regression analyses, is violated [4]. Although the normality assumption is violated by the skewed nature of costs, if the sample size is large enough the central limit theorem ensures that sample means will be normally distributed and standard parametric statistical methods may be used [27]. Log-transformations and standard non-parametric tests (e.g., Mann-Whitney U) are unsuitable for trial-based economic evaluations, since both methods fail to provide an estimate of the mean difference in costs, whereas this is required by decision-makers to allow for estimations of the total budget impact of a new intervention [1, 7, 28, 29]. Suggested methods that are suitable to account for the skewness of costs, while simultaneously comparing mean costs are non-parametric bootstrapping and generalized linear models (GLMs) assuming distributions that fit the data best (e.g. Gamma, Log-Normal and Inverse-Gaussian) [1, 4, 7, 14, 28–32]. An important advantage of non-parametric bootstrapping is that it avoids the need for making distributional assumptions. Of the different non-parametric bootstrapping techniques, Bias-Corrected and Accelerated (BCa) bootstrapping is generally recommended, because it better adjusts for skewness and bias of the sampling distribution, resulting in more accurate confidence intervals than other techniques (e.g. normal bootstrap, percentile bootstrap, bootstrap-t method) [27, 33–35]. GLM can deal with non-normally distributed data as well. However, a choice needs to be made about the most appropriate distribution of the outcome as well as the appropriate link function, which sometimes can be challenging [21, 36]. For the comparison of arithmetic means an identity link could be specified, which obviates the need for complex retransformation techniques [37]. Nevertheless, specification of the identity link is suboptimal as this does not ensure that only positive mean values are estimated as with the Gamma, Log-Normal and Inverse-Gaussian distributions [38].	
*3) Correlated costs and effects*	
Costs and effects are typically correlated and hence their correlation should be accounted for [26, 37, 39]. Proposed methods for dealing with the correlated nature of costs and effects include non-parametric bootstrapping and seemingly unrelated regression (SUR). When resampling cost and effects in pairs, the correlation structure is kept intact when estimating statistical uncertainty [10]. When using SUR, two separate regression models are specified simultaneously; i.e. one for costs and one for effects. In SUR, the correlation between costs and effects is accounted for through correlated error terms [26, 40].	
*4) Missing data*	
In clinical trials, missing data are common [1, 28, 29, 41]. This is of great concern for trial-based economic evaluations, because total costs are calculated as the sum of several cost components measured at different time points. If one resource use item or one time point is missing, total costs will also be missing [1, 28, 29]. According to Rubin [41], missing data can be classified in three mechanisms. First, if missing values are not dependent on any observed or unobserved variable, data are said to be missing completely at random (MCAR). Second, when missing values are related to one or more observed variables, but not the missing value itself, data are said to be missing at random (MAR). Third, when missing data depends on the missing values itself, data are said to be missing not at random (MNAR). In case of MAR and MNAR, bias may be introduced when analyses are restricted to complete cases or when naïve imputation methods, such as mean imputation or last observation carried forward, are used [1, 28, 29, 42]. Only, when missing data can validly be assumed to be MCAR or the proportion of missing data is low (i.e. < 5%), naïve imputation methods may be used [43, 44]. For all other situations, “naïve” imputation methods are discouraged, because - amongst others - they do not account for the uncertainty related to imputing missing values [9, 11, 45–47]. More advanced methods to account for missing data assuming MAR in trial-based economic evaluations include multiple imputation and statistical models with maximum likelihood estimation [9, 11, 45, 46]. Multiple imputation takes into account that imputed values are not the truth and results in valid estimates of mean outcomes and the associated uncertainty [48, 49]. However, multiple imputation heavily relies on correct specification of the imputation model [12]. In addition, with increased complexity of the imputation model, the model might fail to converge [50]. When using maximum likelihood estimation for multilevel missing data, data are not imputed, but all available data is used to compute the maximum likelihood estimate, that is the most likely value had the variable been observed. However, maximum likelihood methods may not be appropriate when observations are missing for multiple variables, which is typically the case for cost data [48, 50].	

## Methods

### Data

To evaluate the impact of whether or not accounting for baseline imbalances, skewed costs, correlated costs and effects, and missing data in trial-based economic evaluations, empirical data from two previously published trial-based economic evaluations were used, the REALISE and HypoAware study.

#### REALISE study

In the Rehabilitation After Lumbar disc Surgery (REALISE) study, early rehabilitation after lumbar disc surgery was compared to no referral after lumbar disc surgery among 169 participants (intervention group: *n* = 92; control group: *n* = 77). Resource use was measured from a societal perspective at 6, 12 and 26 weeks follow-up using cost questionnaires [52]. Resource use was valued using Dutch standard costs [53]. Utility values were based on the EuroQol (EQ-5D-3L), which was administered at baseline and 3, 6, 9, 12 and 26 weeks follow-up [52]. Utility values were estimated using the Dutch tariff for the EQ-5D-3L [54]. Quality-adjusted life years (QALYs) were calculated using linear interpolation between measurement points.

#### HypoAware study

In the HypoAware study, the HypoAware intervention (a blended, group and online psycho-educational intervention based on the evidence-based Blood Glucose Awareness Training) was compared to usual care among 137 participants (intervention group: *n* = 71; control group: *n* = 66) [55]. Resource use was measured from a societal perspective at 2, 4, and 6 months follow-up using cost questionnaires. Utility values were based on the EuroQol (EQ-5D-5L), which was administered at baseline, 2, 4, and 6 months follow-up. Utility values were estimated using the Dutch tariff for the EQ-5D-5L [56]. Quality-Adjusted Life-Years (QALYs) were calculated using linear interpolation between measurement points.

Tables describing baseline characteristics of the REALISE and HypoAware study populations are included in the Appendix (Supplementary Tables 1 and 2). For a detailed description of both studies, the reader is referred elsewhere [52, 55–57].

### Statistical analysis

In total, 14 full economic evaluations were performed for both the REALISE and HypoAware study. In the first analysis, a statistical approach was used, in which baseline imbalances, the skewed nature of cost data, the correlation between costs and effects and missing data were ignored. Thus, this approach simply compared the difference in costs and effects between both groups using t-tests, including only participants with complete cost and effect data, while assuming that both costs and effects were normally distributed and that costs and effects were not correlated. Although this statistical approach ignores all of the challenges in trial-based economic evaluations, it is still being used in practice [7, 8, 10]. Step-by-step, the analyses accounted for the different statistical challenges, until in the final approach all of the statistical challenges were accounted for using the following methods:

- Baseline imbalances: Regression-based adjustment was used [13, 15, 58]. Costs and effects were corrected for their baseline value, if available, and for relevant confounding variables. Variables were considered to be a confounder if the estimated regression coefficients for the cost or effect differences changed by 10% or more when the possible confounding factor was added to the model [58, 59]. For the REALISE study, confounders of costs were participants’ baseline mental health status, physical health status, risk of future work disability, fear-avoidance beliefs about work, treatment credibility and treatment expectations. Confounders of effects included the participants’ baseline utility value, mental health status, back pain, and risk of future work disability. For the HypoAware study, confounders of costs were the participants’ baseline costs, number of severe hypoglycemia episodes during the previous 6 months, and wearing a real-time sensor. Confounders of effects comprised the participants’ baseline utility value and marital status.
- Skewed costs: Non-parametric bootstrapping with 5000 replications was used [4, 7, 33, 40]. The non-parametric bootstrap is a data-based resampling method to estimate statistical uncertainty, without making any distributional assumptions [33]. Bootstrapped confidence intervals were estimated using the bias-corrected and accelerated bootstrap method. The advantage of using bias-corrected and accelerated bootstrapping over percentile bootstrapping, is that it adjusts better for skewness and bias of the sampling distribution, resulting in more accurate confidence intervals [33, 60]. In the REALISE study, the skewness of costs was 1.70 and the kurtosis was 5.75 (excess kurtosis 2.75). In the HypoAware study, the skewness of costs was 1.39 and the kurtosis was 3.90 (excess kurtosis 0.90). The positive skewness indicates that the distribution is skewed to the right and the excess kurtosis indicates a long right tail (i.e. relatively many outliers).
- Correlation between costs and effects: Seemingly unrelated regression (SUR) analysis was used in which two separate regression models were specified simultaneously (i.e. one for costs/one for effects) [26, 40]. In the REALISE study, the correlation between costs and effects was ρ = − 0.42. In the HypoAware study, the correlation between costs and effects was ρ = − 0.44. A negative correlation indicates that individuals with worse outcomes induce higher costs.
- Missing data: Missing data were assumed to be MAR [41]. Multiple Imputation by Chained Equations (MICE) with predictive mean matching (PMM) was used to predict and impute the missing values based on observed data [29, 37]. PMM was used to deal with the skewed distribution of costs [17]. The advantage of PMM is that it is more robust against non-normal data than linear regression estimation methods, as it uses the observed distribution of the data and non-existing values cannot be imputed [61]. As MICE is an iterative procedure, it is important that convergence is reached [17]. This was the case after 100 iterations for the REALISE study and 200 iterations for the HypoAware study. The number of imputed datasets (M) was increased until the loss of efficiency was less than 5%. The loss of efficiency refers to the degree of statistical efficiency of multiple imputation (i.e. accuracy of estimates and confidence interval width) that we are willing to trade-off compared to a situation with an infinite number of imputations and thus maximal efficiency [62]. This resulted in 10 imputed datasets for the REALISE study and 20 imputed datasets for the HypoAware study [63]. The imputed datasets were analysed separately to obtain a set of estimates, which were then pooled using Rubin’s rules [41] to obtain overall estimates, standard errors, and confidence intervals [17, 41, 63]. In the REALISE study, 33 (24%) participants had missing cost data and 21 (15%) had missing effect data. In the HypoAware study, 28 (17%) participants had missing cost data and 20 (12%) had missing effect data.

An overview of the 14 analytical approaches used in this study as well as the statistical challenges they account for can be found in Table 2. For all approaches, incremental costs and QALYs, 95% confidence intervals around incremental costs and QALYs, incremental cost-effectiveness ratios (ICERs) and cost-effectiveness acceptability curves (CEACs) were estimated and compared. ICERs were calculated by dividing incremental mean costs by incremental mean QALYs. CEACs were estimated using the Incremental Net Monetary Benefit (INMB) approach [64]. CEACs represent the probability of an intervention being cost-effective (y-axis) for a range of different ceiling ratios (x-axis) and provide a summary measure of the joint uncertainty surrounding costs and effects [65, 66]. All analyses were performed in StataSE 16® (StataCorp LP, CollegeStation, TX, US).

**Table 2:** Statistical approaches summarized according to the statistical challenge handled

Analysis	Baseline imbalances	Skewed costs	Correlated costs and effects	Missing data
1.- Two t-tests (1 for costs; 1 for effects) complete-case analysis	✗	✗	✗	✗
2.- Two bootstrapped t-tests; complete-case analysis	✗	✓	✗	✗
3.- Two regressions with correction for confounders; complete-case analysis	✓	✗	✗	✗
4.- Two bootstrapped regressions with correction for confounders; complete-case analysis	✓	✓	✗	✗
5.- Two t-tests; mean imputation	✗	✗	✗	✓
6.- Two bootstrapped t-tests; mean imputation	✗	✓	✗	✓
7.- Two regressions with correction for confounders; mean imputation	✓	✗	✗	✓
8.- Two bootstrapped regressions with correction for confounders; mean imputation	✓	✓	✗	✓
9.- Two t-tests; MI	✗	✗	✗	✓
10.- Two bootstrapped t-tests; MI	✗	✓	✗	✓
11.- Two regressions without correction for confounders; MI	✓	✗	✗	✓
12.- Two bootstrapped regression with correction for confounders; MI	✓	✓	✗	✓
13.- SUR without correction for confounders; MI	✗	✓	✓	✓
14.- SUR with correction for confounders; MI	✓	✓	✓	✓

*MI* Multiple Imputation, *SUR* Seemingly Unrelated Regression

✗ - Not accounted for in the analysis

✓ - Accounted for in the analysis

### Comparison of the statistical approaches

Statistical approaches were compared in terms of how sensitive the point estimates are to changes in the statistical approaches (i.e. value sensitivity) and how sensitive the conclusion of an economic evaluation is to changes in statistical approaches (i.e. decision sensitivity) [67]. Value sensitivity was assessed by comparing incremental costs and QALYs, the corresponding confidence intervals, and ICERs across the 14 statistical approaches. Decision sensitivity was assessed by comparing the CEACs of the 14 statistical approaches. For comparing and interpreting the CEACs, thresholds of 0 €/QALY gained, 10,000 €/QALY gained and 23,300 €/QALY gained (i.e. about 20,000 £/QALY gained) were used, which refer to a situation in which decision-makers are not willing to pay anything per QALY gained, the Dutch willingness-to-pay (WTP) thresholds (i.e. between 20,000€/QALY gained and 80,000 €/QALY gained depending on disease severity) and the British National Institute for Health and Care Excellence (NICE) threshold, respectively.

## Results

### Value sensitivity

In the REALISE study, cost and effect differences ranged from -€782 and − 0.001 when ignoring all statistical challenges (analysis 1) to -€82 and 0.011 when accounting for all of them (analysis 14), respectively. The associated ICERs ranged from 636,744 €/QALY gained in analysis 1 to − 7502 €/QALY gained in analysis 14. In analyses 1 to 4, the intervention was less costly and less effective than the control, whereas in analyses 5 to 14 it was less costly and more effective than the control, indicating dominance of the intervention over the control condition. However, in all analyses statistical uncertainty, in terms of confidence interval width, was considerable (see Table 3).

**Table 3:** Results of the cost-effectiveness analyses: REALISE study

Analysis	Societal costs difference (€) (95% CI)	QALY difference (95% CI)	ICER (€/ QALY gained)
1.- Two t-tests (1 for cost; 1 for effects) complete-case analysis	−782 (− 4332 to 2768)	−0.001 (− 0.070 to 0.0677)	636,744
2.- Two bootstrapped t-tests; complete-case analysis	−782 (− 4850 to 2544)	− 0.001 (− 0.070 to 0.0677)	636,744
3.- Two regressions with correction for confounders; complete-case analysis	−197 (− 3692 to 3297)	− 0.009 (− 0.077 to 0.058)	21,390
4.- Two bootstrapped regressions with correction for confounders; complete-case analysis	−197 (− 3643 to 2832)	− 0.009 (− 0.077 to 0.058)	21,390
5.- Two t-tests; mean imputation	− 840 (− 3382 to 1701)	0.004 (− 0.049 to 0.057)	− 231,148
6.- Two bootstrapped t-tests; mean imputation	−840 (− 3467 to 1693)	0.004 (− 0.049 to 0.056)	−231,148
7.- Two regressions with correction for confounders; mean imputation	− 707 (− 3197 to 1783)	0.005 (− 0.047 to 0.057)	− 145,697
8.- Two bootstrapped regressions with correction for confounders; mean imputation	−707 (− 3221 to 1636)	0.005 (− 0.047 to 0.057)	− 231,148
9.- Two t-tests; MI	−141 (− 2318 to 2035)	0.009 (− 0.053 to 0.071)	−16,425
10.- Two bootstrapped t-tests; MI	−141 (− 2398 to 1908)	0.009 (−0.053 to 0.071)	− 16,425
11.- Two regressions with correction for confounders; MI	−73 (− 2295 to 1073)	0.011 (− 0.050 to 0.071)	− 6880
12.- Two bootstrapped regressions with correction for confounders; MI	−73 (− 2248 to 1942)	0.011 (− 0.050 to 0.071)	−6880
13.- SUR without correction for confounders; MI	−141 (− 849 to 1164)	0.009 (− 0.056 to 0.072)	−16,425
14.- SUR with correction for confounders; MI	−82 (− 892 to 1586)	0.011 (− 0.058 to 0.079)	−7502

*95% CI* 95% Confidence Interval, *ICER* Incremental Cost-Effectiveness Ratio, *MI* Multiple Imputation, *SUR* Seemingly Unrelated Regression, *QALY* Quality Adjusted Life-year

In the HypoAware study, cost and effect differences ranged from -€142 and − 0.002 when ignoring all statistical challenges (analysis 1) to -€462 and 0.010 when accounting for all of them (analysis 14), respectively. The associated ICERs ranged from 90,989 €/QALY gained in analysis 1 to 46,592 €/QALY gained in analysis 14. In analyses 1 to 8, the intervention was less costly and less effective than control, in analyses 5 to 8 the intervention was less costly and more effective than the control, indicating dominance of the intervention over the control condition, whereas in analyses 9 to 14 it was more costly and more effective than the control. However, in all analyses statistical uncertainty, in terms of confidence interval width, was considerable (see Table 4).

**Table 4:** Results of the cost-effectiveness analyses: HypoAware study

Analysis	Societal costs difference (€) (95% CI)	QALY difference (95% CI)	ICER (€/ QALY gained)
1.- Two t-tests (1 for cost; 1 for effects) complete-case analysis	−142 (− 1298 to 1014)	−0.002 (− 0.057 to 0.54)	90,989
2.- Two bootstrapped t-tests; complete-case analysis	−142 (− 1326 to 986)	− 0.002 (− 0.057 to 0.54)	90,989
3.- Two regressions with correction for confounders; complete-case analysis	−23 (− 1180 to 1134)	−0.009 (− 0.041 to 0.03)	2536
4.- Two bootstrapped regressions with correction for confounders; complete-case analysis	−23 (− 1170 to 1096)	−0.009 (− 0.041 to 0.03)	2536
5.- Two t-tests; mean imputation	− 105 (− 971 to 759)	0.006 (− 0.037 to 0.049)	− 17,307
6.- Two bootstrapped t-tests; mean imputation	− 105 (− 1032 to 739)	0.006 (− 0.037 to 0.049)	−17,307
7.- Two regressions with correction for confounders; mean imputation	−85 (− 954 to 784)	0.009 (− 0.020 to 0.038)	− 9208
8.- Two bootstrapped regressions with correction for confounders; mean imputation	−85 (− 966 to 770)	0.009 (− 0.020 to 0.038)	− 9208
9.- Two t-tests; MI	438 (− 1033 to 1910)	0.006 (− 0.041 to 0.053)	74,862
10.- Two bootstrapped t-tests; MI	438 (− 940 to 1704)	0.006 (− 0.041 to 0.053)	74,862
11.- Two regressions with correction for confounders; MI	486 (− 995 to 1968)	0.010 (− 0.022 to 0.042)	47,305
12.- Two bootstrapped regressions with correction for confounders; MI	486 (− 904 to 1747)	0.010 (− 0.022 to 0.042)	47,305
13.- SUR without correction for confounders; MI	438 (− 936 to 1712)	0.006 (− 0.042 to 0.053)	74,862
14.- SUR with correction for confounders; MI	462 (− 921 to 1728)	0.010 (− 0.022 to 0.0423)	46,592

*95% CI* 95% Confidence Interval, *ICER* Incremental Cost-Effectiveness Ratio, *MI* Multiple Imputation, *SUR* Seemingly Unrelated Regression, *QALY* Quality Adjusted Life-year

### Decision sensitivity

**Fig. 1:**
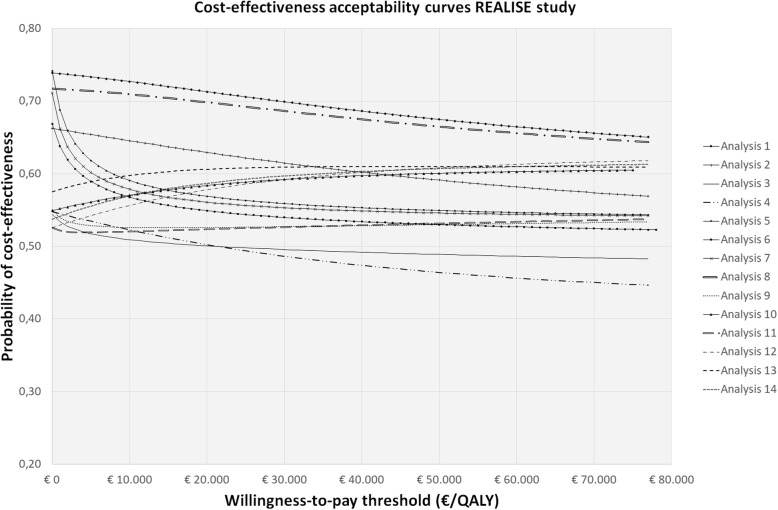
Cost effectiveness acceptability curves indicating the probability of cost-effectiveness at different willingness to pay thresholds in the REALISE study

**Fig. 2:**
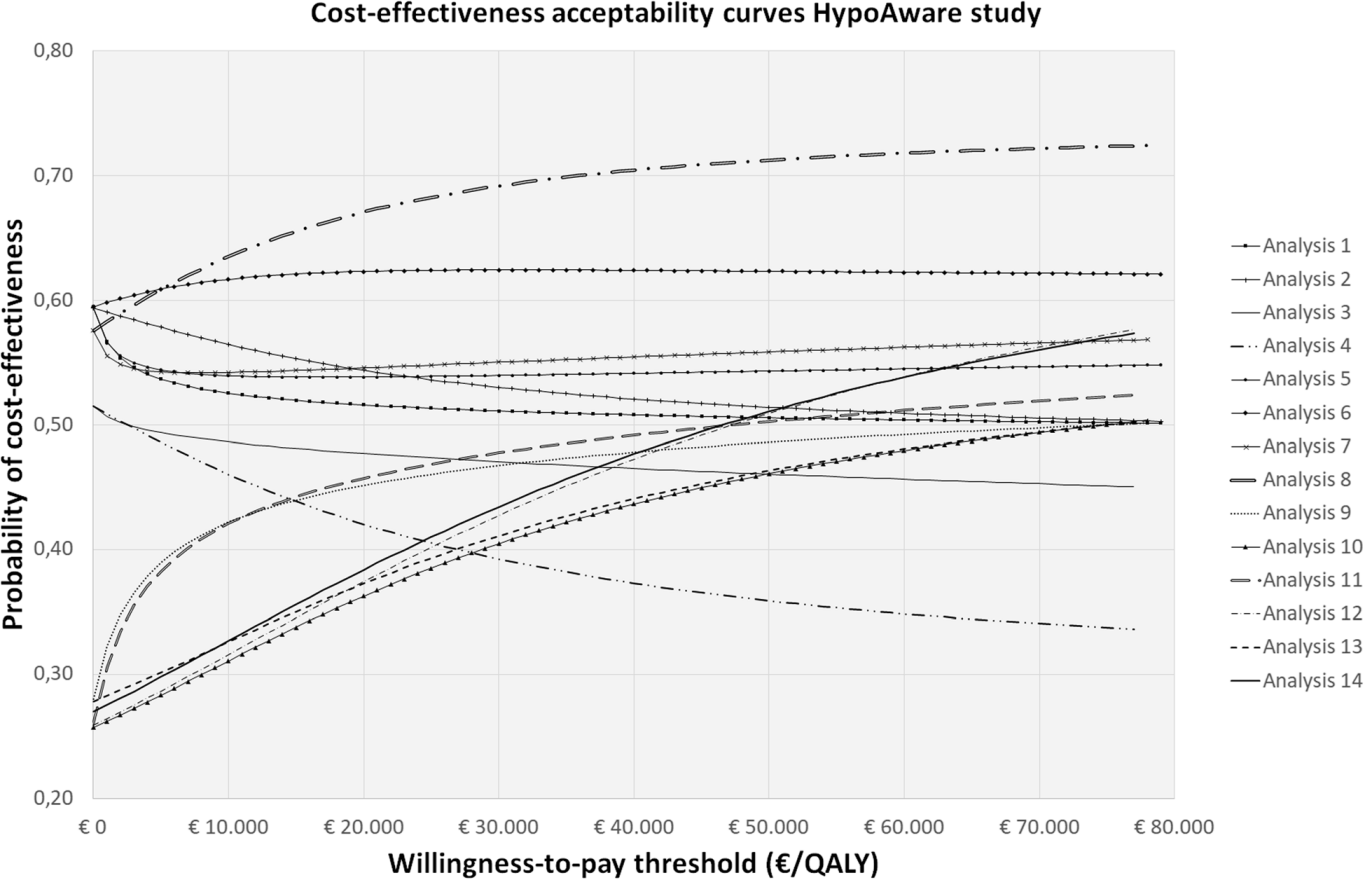
Cost effectiveness acceptability curves indicating the probability of cost-effectiveness at different willingness to pay thresholds in the HypoAware study

In the REALISE study, at a willingness-to-pay of 0 €/QALY, 10,000 €/QALY, and 23,300 €/QALY, the probabilities of cost-effectiveness of the intervention as compared to control were 0.67, 0.57, and 0.55, respectively, when ignoring all statistical challenges (analysis 1) and 0.54, 0.57 and 0.59, respectively, when accounting for all of them (analysis 14). In the HypoAware study, at a willingness-to-pay of 0 €/QALY, 10,000 €/QALY, and 23,300€/QALY, the probabilities of cost-effectiveness of the intervention as compared to control were 0.59, 0.53, and 0.51, respectively, when ignoring all statistical challenges (analysis 1) and 0.27, 0.33, and 0.40, respectively, when accounting for all of them in analysis 14 (Table 5; Figs. 1 and 2).

**Table 5:** Cost–effectiveness probabilities at different willingness to pay thresholds

	REALISE study			HYPOAWARE study		
Analysis	0 €/QALY	10,000 €/QALY	23,300 €/QALY	0 €/QALY	10,000 €/QALY	23,300 €/QALY
1.- Two t-tests (1 for cost; 1 for effects) complete-case	0.67	0.57	0.55	0.59	0.53	0.51
2.- Two bootstrapped t-tests; complete-case	0.66	0.65	0.62	0.59	0.56	0.54
3.- Two regressions with correction for confounders; complete-case	0.54	0.51	0.50	0.52	0.49	0.47
4.- Two bootstrapped regressions with correction for confounders; complete-case	0.54	0.52	0.50	0.52	0.46	0.41
5.- Two t-tests; mean imputation	0.74	0.59	0.57	0.59	0.54	0.54
6.- Two bootstrapped t-tests; mean imputation	0.74	0.73	0.71	0.59	0.62	0.62
7.- Two regressions with correction for confounders; mean imputation	0.71	0.58	0.56	0.58	0.54	0.55
8.- Two bootstrapped regressions with correction for confounders; mean imputation	0.72	0.71	0.69	0.58	0.64	0.68
9.- Two t-tests; MI	0.55	0.53	0.53	0.28	0.42	0.45
10.- Two bootstrapped t-tests; MI	0.55	0.57	0.59	0.26	0.31	0.38
11.- Two regressions with correction for confounders; MI	0.53	0.52	0.53	0.26	0.42	0.46
12.- Two bootstrapped regression with correction for confounders; MI	0.53	0.56	0.58	0.26	0.32	0.40
13.- SUR with correction for confounders; MI	0.58	0.60	0.61	0.28	0.33	0.39
14.- SUR with correction for confounders; MI	0.54	0.57	0.59	0.27	0.33	0.40

*MI* Multiple Imputation, *SUR* Seemingly Unrelated Regression

## Discussion

The current findings indicate that failure to adequately account for baseline imbalances, skewed costs, correlated costs and effects, and missing data in trial-based economic evaluations may have a substantial impact on cost-effectiveness results.

Correction for baseline imbalances showed to have a large impact on the point estimates for both costs and effects, with the impact being most pronounced for costs. When accounting for skewed costs using bootstrapping, in some cases the estimated statistical uncertainty (i.e. confidence intervals) around cost differences increased. However, for the majority of the statistical approaches, the confidence interval width was relatively similar between bootstrapped and non-bootstrapped statistical approaches. Taking into account the correlation between costs and effects had no large effects on the point estimates, nor on the statistical uncertainty surrounding both outcomes. When using different methods to account for missing data, point estimates as well as the amount of statistical uncertainty differed considerably between analyses. These methods consequently had the largest impact on the probabilities of the interventions being cost-effective compared with the control.

Overall, these results indicate that point estimates as well as statistical uncertainty reflected in the probabilities of cost-effectiveness are notably affected when adjusting for baseline imbalances and missing data in trial-based economic evaluations. Although the impact of adjusting for skewness and the correlation between costs and effects was limited, the presence of all four statistical challenges in the data indicates that a statistical approach that takes into account all of these challenges simultaneously was the most appropriate approach and is expected to lead to the most valid results and conclusions.

### Strengths and limitations

The current study is the first to systematically evaluate the impact of simultaneously adjusting for baseline imbalances, skewed costs, correlated costs and effects, and missing data in trial-based economic evaluations on cost differences, effect differences, ICERs, and statistical uncertainty. Another strength is that, step-by-step, all statistical challenges were accounted for until all statistical challenges were dealt with, thus also showing the separate impact of accounting for each of these challenges. Finally, all of the applied statistical methods have previously been found to be valid (see references in Table 1). The main limitation of this study is generalizability, as the findings likely depend on the characteristics of the datasets that were analyzed. In addition, the applied statistical methods were employed from a frequentist approach. Bayesian methods are generally more flexible and the interpretation of their results is more intuitive than those of frequentist methods [68–72]. However, Bayesian methods are generally more complex to implement and are less commonly known to most healthcare researchers. Therefore, in line with Gomes et al. [73] we think that frequentist approaches are more likely to improve current practice. Another limitation is that the true outcomes of the REALISE and HypoAware study are not known. Therefore, the performance of the combination of statistical methods used in this study could not be assessed. This prevents providing a concrete recommendation about which analytical approach is best. For a concrete recommendation about which statistical method is most appropriate, simulated data would be needed, where the true parameters are known and methods can be evaluated in terms of how close their estimates are to the pre-specified parameters (i.e. empirical bias), how well the methods fit the data (i.e. root mean squared error) and whether they result in valid estimates of uncertainty (i.e. coverage probability) [74]. Finally, although we found a considerable impact of using either one of the statistical approaches on the probabilities of cost-effectiveness in both studies, the conclusions of the studies did not change. Nonetheless, it can be easily imagined that a difference of 0.20 in the probability of cost-effectiveness between analyses 1 and 14 at a ceiling ratio of 10,000 €/QALY (HypoAware study) can lead to a different conclusion in other situations.

### Comparison to other studies and implications for further research and practice

One can argue that it is already known that when using different statistical approaches, different results will be obtained. The results of this study reinforce this message and show that it is of utmost importance to align the statistical approach with the statistical challenges identified in a specific dataset. Nonetheless, current practice still shows discrepancies between the statistical approach used and the statistical challenges present in a dataset. For instance, missing data and skewed costs are still inappropriately handled in many trial-based economic evaluations [7, 8, 10], but can have a large impact on the results as illustrated by this study. Amongst others, failure to appropriately handle these issues may be due to a lack of consensus about what the most optimal methods for dealing with baseline imbalances, skewed costs, correlated costs and effects, and missing data in trial-based economic evaluation are.

Based on the recommendations of several simulation studies within the literature, the statistical methods used in this study are currently considered amongst the most appropriate methods (see Table 1). However, statistical methods are in continuous development. For example, multiple imputation is nowadays generally recommended to deal with missing data [8, 12, 17, 41, 51, 75]. However, Twisk et al. [76] showed that multiple imputation was not necessary when using longitudinal mixed model analyses to estimate clinical effects, although it is unclear whether this also holds for cost-effectiveness data. Additionally, missing data was assumed to be MAR, however, this assumption might not always hold and data could be MNAR. Recently, an increasing number of guidelines and studies emphasize the importance of checking for possible departure from the MAR assumption [12, 31, 42, 77, 78]. It is recommended to perform sensitivity analyses, using other methods such as selection and/or pattern-mixture models [77]. Furthermore, the handling of clustered data or longitudinal data was not investigated in this study, whereas failure to account for clustering will underestimate statistical uncertainty, can lead to inaccurate point estimates, and may in turn lead to incorrect inferences [10, 71, 79]. It is also important to note that the statistical challenges identified in this study are only a selection of possible statistical issues that might arise when analyzing trial-based economic evaluations [51]. Therefore, in order to improve the statistical quality of trial-based economic evaluations, it is helpful to expand the health economic literature by laying out the current state of play regarding statistical methods for trial-based economic evaluations, and to develop guidance and frameworks in which specific statistical methods are recommended to be used in trial-based economic evaluations.

## Conclusion

The current study emphasizes the importance of adequately accounting for baseline imbalances, skewed costs, correlated costs and effects, and missing data in trial-based economic evaluations by demonstrating that ignoring them may lead to different cost-effectiveness results. Therefore, when conducting trial-based economic evaluations, it is of utmost importance to first check the data to identify statistical challenges that need to be accounted for in the analysis, and then adequately deal with them. Furthermore, it is worthwhile to develop consensus among researchers about frameworks and guidelines on how to best analyze trial-based economic evaluations. To facilitate researchers in appropriately dealing with baseline imbalances, skewed costs, correlated costs and effects, and missing data in trial-based economic evaluations, a software code (Stata®) is provided in Supplementary File 1 for the most advanced statistical approach.

## Supplementary Information

**Additional file 1: Supplementary Table 1.** Baseline characteristics REALISE study.

**Additional file 2: Supplementary Table 2.** Baseline characteristics HypoAware study.

**Additional file 3.** Stata® syntax.

## Data Availability

The datasets used and/or analysed during the current study available from the corresponding author on reasonable request.
