# Supplementary material for: The statistical approach in trial-based economic evaluations matters: get your statistics together!
Source: BMC Health Serv Res. 2021 May 19;21:475. doi: 10.1186/s12913-021-06513-1 (PMC8135982; doi:10.1186/s12913-021-06513-1)
Supplement: Supplementary file 1 — Additional file 1: Supplementary Table 1. Baseline characteristics REALISE study. [file 12913_2021_6513_MOESM1_ESM.docx]

SUPPLEMENTARY TABLE 1. Baseline characteristics REALISE study.

| **Baseline characteristics** | **Control group (SE)** *(n=77)* | **Intervention group (SE)** *(n=92)* |
| --- | --- | --- |
| Age in years | 46.7 (12.2) | 46.9 (11.6) |
| Female (n, %) | 44 (57.1) | 54 (58.7) |
| Living alone (n, %) | 8 (10.4) | 16 (16.3) |
| Education (n, %) |  |  |
| *Low* | 17 (22.1) | 20 (21.8) |
| *Middle* | 35 (45.5) | 47 (51.1) |
| *High* | 25 (32.5) | 25 (27.1) |
| Employment (n, % yes) | 57 (74.0) | 74 (80.4) |
| Level of herniation (n, %) |  |  |
| *L2-3* | 2 (2.6) | 1 (1.1) |
| *L3-4* | 4 (5.2) | 10 (10.9) |
| *L4-5* | 42 (58.3) | 31 (33.7) |
| *L5-S1* | 29 (37.7) | 48 (52.2) |
| *L5-6* | 2 (2.6) | 1 (1.1) |
| Type of herniation (n, %) |  |  |
| *Sequester* | 34 (44.2) | 34 (37.0) |
| *Bulging disc* | 46 (59.7) | 57 (62.0) |
| *Extraforaminal* | 2 (2.6) | 1 (1.1) |
| Functional status (ODI, 0-100) | 50.4 (15.6) | 48.6 (17.3) |
| Pain intensity leg (NRS, 0-10)) | 7.7 (1.8) | 7.8 (1.9) |
| Pain intensity back (NRS, 0-10)) | 6.1 (2.6) | 6.5 (2.5) |
| General physical health (SF12, 0-100) | 26.7 (15.4) | 26.2 (16.1) |
| General mental health (SF12, 0-100) | 50.3 (21.8) | 51.6 (21.5) |
| Psychosocial status (OMPSQ, 0-210) | 114.2 (20.5) | 109.0 (24.9) |
| Fear avoidance beliefs physical activity (FABQ, 0-24) | 15.4 (5.4) | 16.1 (4.4) |
| Fear avoidance beliefs work (FABQ) | 18.5 (11.3) | 16.8 (11.0) |
| Expectation: expectancy surgery (CEQ, 3-27) | 22.9 (3.0) | 23.2 (2.8) |
| Expectations : credibility surgery (CEQ, 3-27)) | 21.7 (3.7) | 22.0 (3.2) |
| Expectations: credibility item intervention (CEQ 1-9) | 6.3 (1.8) | 6.5 (1.8) |
| Expectations: credibility item control (CEQ 1-9) | 6.5 (1.4) | 6.4 (1.6) |
| Pain Coping : active (PCI) | 6.5 (1.3) | 6.7 (1.3) |
| Pain Coping: passive (PCI) | 6.5 (1.2) | 6.5 (1.3) |
| Duration of complaints (n, %) |  |  |
| *0-1months* | 0 | 2 (2.2) |
| *1-2 months* | 3 (3.9) | 6 (6.6) |
| *2-3 months* | 7 (9.1) | 1 (1.1) |
| *3-6 months* | 29 (37.7) | 35 (38.0) |
| *6-9 months* | 13 (16.9) | 18 (19.6) |
| *9-12 months* | 7 (9.1) | 6 (6.5) |
| *> 12 months* | 18 (23.4) | 24 (26.1) |
| Medication use (n, %) |  |  |
| *Every day* | 47 (61.0) | 56 (60.9) |
| *Not every day* | 14 (18.2) | 18 (19.6) |
| *No* | 16 (20.8) | 18 (19.6) |
| Surgical complications (n, %) |  |  |
| *Nerve root injury* | 1 (1.3) | 1 (1.1) |
| *Dural tear* | 2 (2.6) | 2 (2.2) |
| *Increase in sensimotor deficit* | 1 (1.3) | 0 |
