# Supplementary material for: The statistical approach in trial-based economic evaluations matters: get your statistics together!
Source: BMC Health Serv Res. 2021 May 19;21:475. doi: 10.1186/s12913-021-06513-1 (PMC8135982; doi:10.1186/s12913-021-06513-1)
Supplement: Supplementary file 2 — Additional file 2: Supplementary Table 2. Baseline characteristics HypoAware study. [file 12913_2021_6513_MOESM2_ESM.docx]

SUPPLEMENTARY TABLE 2. Baseline characteristics HypoAware study.

| **Baseline characteristics** | **Control group (SE) (n=66)** | **Intervention group (SE) (*n* =71)** |
| --- | --- | --- |
| Age (years) | 51.3 (14.0) | 52.7 (12.4) |
| Female, *n* (%) | 29 (44) | 34 (48) |
| BMI | 25.2 (3.9) | 26.0 (5.3) |
| Dutch origin, *n* (%) | 65 (99) | 67 (95) |
| Employed, *n* (%) | 37 (56) | 37 (52) |
| Education, *n* (%) |  |  |
| Primary education | 16 (24) | 23 (32) |
| Secondary education | 30 (46) | 20 (28) |
| Higher education | 20 (30) | 28 (39) |
| With partner, *n* (%) | 46 (70) | 53 (75) |
| Type diabetes, *n* (%) |  |  |
| Type 1 diabetes, | 59 (91) | 62 (87) |
| Type 2 diabetes | 6 (9) | 8 (11) |
| Other (MODY) | 1 (2) | 1 (1) |
| Treatment, *n* (%) |  |  |
| CSII | 36 (55) | 29 (41) |
| MDI | 30 (46) | 42 (59) |
| Comorbidity, *n* (%) | 33 (50) | 40 (56) |
| HbA_1c_ (mmol/mol) | 60.4 (12.2) | 60.8 (11.2) |
| HbA_1c_ (%) | 7.7 (1.1) | 7.7 (1.0) |
| Diabetes duration (years) | 27.5 (13.1) | 24.6 (14.0) |
| Age of diagnosis (years) | 23.9 (12.2) | 28.2 (13.9) |
| Complications, *n* (%) | 28 (42) | 29 (41) |
| Attended a diabetes education program, *n* (%) | 10 (15) | 8 (11) |
| Previous experience with real-time sensor, *n* (%) | 19 (29) | 23 (32) |
| Number of blood glucose measurements per day | 4.5 (2.2) | 4.6 (2.3) |
| Non-severe hypoglycemic events per week (,4 mmol/L [,72 mg/dL])* | 7.4 (3.9) | 5.3 (3.8) |
| Impaired hypoglycemia awareness (Gold score), *n* (%) | 48 (73) | 56 (79) |
| Severe hypoglycemic events in the previous 6 months | 1 (0–5) | 2 (0–6) |
| Data are reported as the mean (SD) or median (IQR), unless otherwise indicated. CSII, continuous subcutaneous insulin infusion; MDI, multiple daily injections. *In participants without RT-CGM at T1 (total *n* = 98; control *n* = 45; intervention *n* = 53). | | |
