# Supplementary material for: The statistical approach in trial-based economic evaluations matters: get your statistics together!
Source: BMC Health Serv Res. 2021 May 19;21:475. doi: 10.1186/s12913-021-06513-1 (PMC8135982; doi:10.1186/s12913-021-06513-1)
Supplement: Supplementary file 3 — Additional file 3. Stata® syntax. [file 12913_2021_6513_MOESM3_ESM.docx]

**The statistical approach in trial-based economic evaluations matters: get your statistics together!**

By:

*Elizabeth N. Mutubuki,^1,4^ *Mohamed El Alili,^2^ Judith E. Bosmans,^2^  Teddy Oosterhuis,^2^ Frank Snoek,^3^ Raymond W. J. G. Ostelo,^1,4^ Maurits W. van Tulder,^5,6^ Johanna M. van Dongen,^1,2^

*Contributed equally

Journal: BMC Health Services Research

^1^Department of Health Sciences, Faculty of Science, VU Amsterdam, Amsterdam Movement Sciences Research Institute, the Netherlands

^2^Department of Health Sciences, Faculty of Science, VU Amsterdam, Amsterdam Public Health Research Institute, the Netherlands

^3^Department of Medical Psychology, Amsterdam UMC, VU Amsterdam, Amsterdam, the Netherlands

^4^Department of Epidemiology and Biostatistics, Amsterdam UMC, Location VUmc, Amsterdam Movement Sciences Research Institute, the Netherlands

^5^Department of Physiotherapy & Occupational Therapy, Aarhus University Hospital, Aarhus, Denmark

^6^Department of Human Movement Sciences, Faculty Behavioural and Movement Sciences, Vrije Universiteit Amsterdam

Corresponding author:

Mohamed El Alili, Department of Health Sciences, Faculty of Science, VU Amsterdam, De Boelelaan 1085, 1081 HV, Amsterdam, the Netherlands. Tel.: +31 20 59 82790; Fax: +31 20 6462457; E-mail: [m.elalili@vu.nl](mailto:m.elalili@vu.nl)

Of note: Please refer to this study when using the below syntax.

*******MULTIPLE IMPUTATION**********

clear

set more off

cd "XXX"

capture log close

log using "XXXX.smcl", replace

use “XXXXXX.dta”, clear

misstable summarize AAAA BBBB CCCC DDDD EEEE, gen(M_)

mi set flong

mi register regular AAAA BBBB CCCC DDDD

mi register imputed EEEE FFFF GGGG HHHH

mi impute chained (pmm, knn(XXXX)) EEEE FFFF GGGG HHHH = AAAA BBBB CCCC DDDD , by(XXXX) add(XX) replace rseed(XXXX)

save "XXXX.dta ", replace

* Save imputated datasets seperately *

local n = XXX

forvalues j=1(1)`n' {

use "XXXXXX.dta", clear

keep if _mi_m ==`j'

save "XXXXXX `j'", replace

}

*

capture log close

********** CEA ANALYSES **********

clear

set more off

cd "XXX"

capture log close

log using "XXXX.smcl", replace

***** Fill in n = number of imputations *****

local n = XXX

forvalues j=1(1)`n' {

local y = `j'

use "XXXXX`y'", clear

bootstrap bootcost_diff = _b[YYYY:XXXX] booteffect_diff = _b[YYYY:XXXX], reps(XXXX) seed(XXXX) saving("boots`y'", replace) bca: sureg (YYYY = XXXX) (YYYY = XXXX)

mat betaCE= e(b) /* extract the matrix of regression coefficients */

mat se = e(se) /* extract standard errors */

mat limits = e(ci_bc) /* extract confidence limits */

mat vari = e(V) /* extract the variance-covariance matrix */

gen cost_diff = betaCE[1,1] /* create differential costs */

gen effect_diff = betaCE[1,2] /* create differential effects */

gen N = e(N) /* extract sample size*/

gen LL_effect = limits[1,2]

gen UL_effect = limits[2,2]

gen LL_cost = limits[1,1]

gen UL_cost = limits[2,1]

gen cost_var = vari[1,1] /* extract the variance of the mean differential costs from the VC matrix */

gen effect_var = vari[2,2] /* extract the variance of the mean differential effect from the VC matrix */

gen cov = vari[1,2] /* extract the covariance between mean differential costs and effect */

save postboots`y', replace

}

clear

set more off

cd "XXXX"

***** Fill in n = number of imputations *****

local n = XX

/* append bootstrap samples in 1 file */

use boots1, clear

forvalues k=2(1)`n' {

local z = `k'

append using boots`z'

}

save boots, replace

*** All information from the extra information from bivariate regression needs to be appended, allowing to pool according to Rubin’s rules ***

use postboots1, clear

forvalues l=2(1)`n' {

local a = `l'

append using postboots`a'

}

by _mi_m, sort: drop if _n != _N

save postboots, replace

keep cost_diff LL_cost UL_cost cost_var effect_diff LL_effect UL_effect effect_var cov

append using boots

gen Za=1.95996

/* estimate confidence limits for effects using Rubin's rules */

egen effect_diff_pooled = mean(effect_diff)

egen W=mean(effect_var)

gen _Bdiff=(effect_diff-effect_diff_pooled)^2

egen _Bsum=total(_Bdiff)

gen B=(1/(`n'-1))*_Bsum

gen T=W+(1+(1/`n'))*B

gen seT=sqrt(T)

gen LL_effect_pooled=effect_diff_pooled -(Za*seT)

gen UL_effect_pooled=effect_diff_pooled +(Za*seT)

/* estimate bias-corrected and accelerated confidence limits for costs */

egen cost_diff_pooled = mean(cost_diff)

egen LL_cost_pooled = mean(LL_cost)

egen UL_cost_pooled = mean(UL_cost)

generate ICER = cost_diff_pooled /effect_diff_pooled

display ICER

display effect_diff_pooled

display LL_effect_pooled

display UL_effect_pooled

display cost_diff_pooled

display LL_cost_pooled

display UL_cost_pooled

label variable bootcost_diff "Bootstrapped estimates"

label variable cost_diff_pooled "Point estimate"

twoway (scatter bootcost_diff booteffect_diff, msize(small)) (scatter cost_diff_pooled effect_diff_pooled, msize(small)), ///

ytitle(Cost differences (€)) yline(0) xline (0) ///

name(CEplane, replace)

graph save "CEplane.gph", replace

gen quadrantcompl1 = 0

replace quadrantcompl1 = 1 if bootcost_diff > 0 & booteffect_diff > 0

replace quadrantcompl1 = 2 if bootcost_diff < 0 & booteffect_diff > 0

replace quadrantcompl1 = 3 if bootcost_diff < 0 & booteffect_diff < 0

replace quadrantcompl1 = 4 if bootcost_diff > 0 & booteffect_diff < 0

label variable quadrantcompl1 "quadrant of CE plane"

label define quadrantcompl1 1 NEQuadrant 2 SEQuadrant 3 SWQuadrant 4 NWQuadrant

sort quadrantcompl1

proportion quadrantcompl1

/* estimate CEA curve using Rubin's rules */

forvalues i= 0 (1000) 80000 { /* local macro i counts from 0 to 80000 in steps of 1000 */

local x = `i'/ 1000 /* x is created just for variable names */

gen NB`x'=(`i'*effect_diff)-cost_diff /* NBs are generated for each value of i */

gen varNB`x'=`i'^2 * effect_var + cost_var - 2*`i'*cov /* variance of NB is generated */

gen seNB`x'=sqrt(varNB`x') /* standard error of NB is generated */

egen meanNB`x'=mean(NB`x')

egen W_NB`x'=mean(varNB`x')

gen _Bdiff_NB`x'=(NB`x'-meanNB`x')^2

egen _Bsum_NB`x'=total(_Bdiff_NB`x')

gen B_NB`x'=(1/(`n'-1))*_Bsum_NB`x'

gen T_NB`x'=W_NB`x'+(1+(1/`n'))*B_NB`x'

gen seT_NB`x'=sqrt(T_NB`x')

local z = meanNB`x'/seT_NB`x'

local prob = normal(`z')

matrix row = (`i',`prob')

matrix ceac = (nullmat(ceac)\row) /* Matrix containing probability that intervention is cost-effective for each value of i */

}

svmat ceac /* The matrix is converted into variables */

matrix drop ceac /* The unneeded matrix is now dropped */

twoway (line ceac2 ceac1), ytitle(Probability intervention cost-effective) yscale(range(0 1)) ylabel(0 (0.2) 1) xtitle(Ceiling ratio: €/ QALY) xscale(range(0 80000)) xlabel(0 (10000) 50000)

graph save "CEAC.gph", replace

save postboots, replace

capture log close
